# Guideline for the analysis of the microbial communities of the human upper airways

**DOI:** 10.1080/20002297.2022.2103282

**Published:** 2022-07-28

**Authors:** Leonardo Mancabelli, Tecla Ciociola, Gabriele Andrea Lugli, Chiara Tarracchini, Federico Fontanta, Alice Viappiani, Francesca Turroni, Andrea Ticinesi, Tiziana Meschi, Stefania Conti, Marco Ventura, Christian Milani

**Affiliations:** a Department of Chemistry, Life Sciences and Environmental Sustainability, University of Parma, Parma, Italy; b Department of Medicine and Surgery, University of Parma, Parma, Italy; c GenProbio srl, Parma, Italy; d Microbiome Research Hub, University of Parma, Parma, Italy; e Geriatric-Rehabilitation Department, Azienda Ospedaliero-Universitaria di Parma, Parma, Italy

**Keywords:** Metagenomics, shallow shotgun, microbiota, microbiome

## Abstract

The recent COVID-19 pandemic prompted a rapid-growing interest in the investigation of the human microbiota of the upper airways. In fact, the resident microbial community of this body district may have an influence on the onset of SARS-CoV-2 infection and its clinical course in terms of presence, symptom severity, and outcomes. However, several microbiological methodologies are available to study the human microbiota, reflecting the extensive fragmentation of methodological approaches. We investigate the impact of two critical steps that can induce biases in the downstream analyses, i.e. sampling method and microbial DNA extraction kit employed. We observed major discrepancies regarding the total amount of prokaryotic DNA that could be retrieved from a biological sample and the proportion between bacterial DNA and human host DNA. Moreover, shotgun DNA sequencing and taxonomic profile reconstruction also revealed correlations between sampling methods and the procedures applied for microbial DNA extraction. Based on all the data collected in this study, we formulate indications regarding the most efficient and reliable methodological procedures for the metagenomic analyses of the upper airways’ microbiota to maximize accuracy and reproducibility.

## Introduction

The respiratory tract harbors a complex community of microorganisms that establish a symbiotic relationship with the host [1]. These microorganisms, known as microbiota, may contribute to preventing respiratory pathogens colonization, in the maturation of the respiratory tract [2], and in shaping local immunity [3–5]. Moreover, the respiratory microbiota was reported to play a crucial role as a barrier to bacterial and/or viral infections [1,5]. In this context, many recent studies have suggested a possible correlation with the current COVID-19 pandemic [6–10]. The respiratory microbiota may in fact be involved in the onset of bacterial superinfection occurring in the advanced phases of severe symptomatic forms of COVID-19, particularly in those patients who require invasive ventilator support in intensive care units (ICUs) for respiratory failure [11,12]. Furthermore, recent metagenomic studies reported a substantial alteration in oral [7] and oropharyngeal [13] microbiota of COVID-19 patients compared to healthy controls. In detail, COVID-19 patients showed a decrease in bacterial biodiversity, suggesting an association between the microbiome community complexity and the disease severity [13]. Moreover, the alteration of the respiratory microbiota, corresponding to an increase in opportunistic pathogens, could contribute to the severity of COVID-19 infection and represent a predictor of clinical outcomes, including the need for ventilator support and mortality [14].

The metagenomic approaches, such as 16S rRNA gene profiling and shotgun/shallow shotgun metagenomics, allowed us to investigate in depth the composition of the human microbiota, particularly of the human respiratory tract [1,15–17]. Despite the many advantages of metagenomic approaches, sample collection and DNA extraction remain the major biases for obtaining reliable results [18–21]. In fact, several studies reported that the collection method of biological samples might significantly impact the results of human microbiota analysis [18,22]. Moreover, microbial DNA extraction represents a crucial step in achieving high-quality prokaryotic DNA, allowing accurate profiling of the microbiota composition of the biological sample assayed [22–24]. Thus, the use of different microbial DNA extraction protocols based on different commercially available kits could strongly affect the determined bacterial composition.

In this study, we tested the performances of the most widely applied microbial sampling protocols employed in respiratory tract-related studies and the main different commercially available microbial DNA extraction kits used for metagenomic approaches, including shotgun metagenomics.

## Materials & methods

### Samples collection

The samples were collected from seven adults without respiratory symptoms (Table S3). This study was approved as part of a larger project on the study of respiratory microbiome in COVID-19 by the local Ethics Committee (Comitato Etico dell’Area Vasta Emilia Nord, Emilia-Romagna Region, Italy), under the ID 1131/2020/TESS/UNIPR.

Nasopharyngeal and oropharyngeal swabs were collected according to standard procedures (https://www.cdc.gov/) with FLOQSwabs® (COPAN). After collection, swabs were inserted into tubes containing 1.5 mL of inactivating DNA/RNA shield buffer (Zymo Research, USA). Saliva samples were collected into 2 mL collection tubes.

### DNA extraction

The samples were processed immediately after collection. In particular, 1.2 mL of each sample was transferred into a 1.5 mL collection tube, centrifugated at 16,000 × g for 3 min, and the supernatant discarded. The subsequent DNA extraction was performed following the manufacturer’s instructions for each DNA extraction kit.

For QIAmp DNA Mini Kit, the procedure has been partially modified in order to optimize the extraction. Briefly, for swab samples, the pellet obtained in the previous step was resuspended in 600 μL PBS and transferred into glass bead tube; then, the sample was subjected to three 2 min pulses at maximum speed in a bead beater with intervals of 2 min on ice before proceeding with the manufacturer’s instructions. For saliva samples, the pellet obtained in the previous step was resuspended in 100 μL Buffer TE and, after that, 180 μL Buffer ATL and 20 μL proteinase K were added; the sample was mixed immediately by vortexing at maximum speed and incubated at 56°C for 90 min. During incubation, the sample was mixed occasionally. Subsequently, 200 μL Buffer AL was added to the sample, mixed again by vortexing and incubated at 70°C for 10 min. Then, 200 μL ethanol (96–100%) was added to the sample before proceeding with the manufacturer’s instructions.

DNA samples were stored at −20°C until use.

### Mock community

Well-known bacterial reference strains and clinical isolates were used in this study (Table 1). *Klebsiella pneumoniae* ATCC 700603, *Pseudomonas aeruginosa* ATCC 9027, *Staphylococcus aureus* ATCC 29213, *Stenotrophomonas maltophilia* ATCC 17666, *Streptococcus mutans* UA159, and *Streptococcus pneumoniae* ATCC 6301 were the reference strains. The clinical isolates (*Acinetobacter baumannii, Pseudomonas spp., Staphylococcus haemolyticus*, and *Streptococcus pneumoniae*), some of which have been described in previous studies [25,26], derived from the collection of Microbiology and Virology Laboratory, University of Parma, Italy. A microscopic counting after Gram staining was performed for each bacterial suspension. To prepare the mock communities, bacterial suspensions were mixed and properly diluted to obtain a final concentration of 10^6^ bacterial cells (Table 2). Furthermore, two additional bacterial mock communities at concentration of 10^6^ and 10^4^ bacterial cells were prepared with the inclusion of 10^6^ eukaryotic cells (ATCC CCL-171™, MRC-5 human fibroblast cell line derived from normal lung tissue).

**Table 1.** Bacteria included in the mock community.

Species		Number of strains
*Acinetobacter baumannii* 2	Clinical isolate [25]	1
*Klebsiella pneumoniae* ATCC 700603	Reference strain	1
*Pseudomonas aeruginosa* ATCC 9027	Reference strain	1
*Pseudomonas* spp. 1014, 1017, 1023, 1025	Clinical isolates*	4
*Staphylococcus aureus* ATCC 29213	Reference strain	1
*Staphylococcus haemolyticus* 221–4**, SH8	Clinical isolates [26]	2
*Stenotrophomonas maltophilia* ATCC 17666	Reference strain	1
*Streptococcus mutans* UA159	Reference strain	1
*Streptococcus pneumoniae* ATCC 6301	Reference strain	1
*Streptococcus pneumoniae* 143, 153	Clinical isolates [26]	2

*from the collection of Microbiology and Virology Laboratory, University of Parma, Italy.

**stable teicoplanin-resistant clone obtained in population studies from heterogeneously teicoplanin-susceptible clinical isolates.

**Table 2.** Heatmap reports the taxonomical composition of the expected mock (mix-Ex) and the mock communities treated with different DNA extraction kits. Moreover, the percentage relative change between mix-Ex and each specific treated mock is reported.

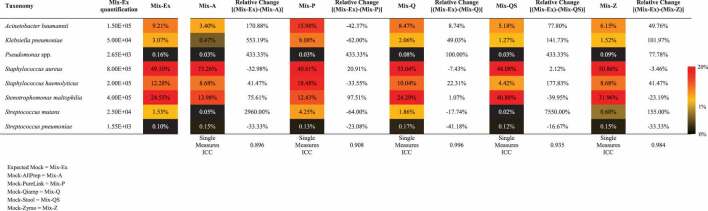

Expected Mock = Mix-Ex

Mock-AllPrep = Mix-A

Mock-PureLink = Mix-P

Mock-Qiamp = Mix-Q

Mock-Stool = Mix-QS

Mock-Zymo = Mix-Z

### Shallow shotgun sequencing

A DNA library was prepared using the Nextera XT DNA sample preparation kit (Illumina, San Diego, California, USA) according to the manufacturer’s instructions. In detail, one ng input DNA from each sample was used for library preparation. The isolated DNA underwent enzymatic fragmentation, adapter ligation, and purification involving magnetic beads.

Then, samples were quantified using a fluorometric Qubit quantification system (Life Technologies, Thermo Fisher Scientific, Waltham, Massachusetts, USA) loaded on a 2200 Tape Station Instrument (Agilent Technologies, Santa Clara, California, USA) and normalized to 4 nM. Sequencing was performed on a MiSeq instrument (Illumina, San Diego, California, USA), according to the manufacturer’s instructions, using the 2 × 250 MiSeq Reagent Kit v3 (600-cycle), and spike-in of 1% PhiX control library.

### Shallow taxonomic profiling

Taxonomic profiling of sequenced reads was performed with the METAnnotatorX2 bioinformatics platform (Computational Microbiology Unit, University of Parma, Parma, Italy) [27]. In detail, the raw data in fastq format were submitted to quality filtering with removal of reads with an average quality <25. Subsequently, host DNA was removed by reads mapping to the human genome. Retained sequences were used as input to perform a MegaBLAST local alignment of reads to a pre-processed database, including available genomes of eukaryotes (Fungi and Protists), bacteria, archaea, and viruses, following the METAnnotatorX2 manual [27]. Reads showing a nucleotide identity >94% to the genomes included in the database were classified at the species level, while if a lower percentage identity was detected, they were classified at the genus level as undefined species. These cut-offs are those generally employed for the ANI taxonomic assignment of genomes.

### Statistical analysis

ORIGIN 2021 (https://www.originlab.com/2021) and SPSS software (www.ibm.com/software/it/analytics/spss/) were used to compute statistical analyses. EMPeror tool was used to visualize PCoA analyses [28] calculated through ORIGIN 2021. Furthermore, comparisons between groups were tested by t-test analysis. Intraclass correlation (ICC) analysis was used to compare the mock communities’ taxonomical profiles.

## Data availability

The BioProject accession number of the metagenomic sequences obtained in this study is PRJNA786898.

### Results

#### Evaluation of the performances of DNA extraction kits for respiratory tract-related microbiota analysis employing artificial microbial community

Amongst the most critical steps in delineating the composition of the microbial community residing in a human-body site, the DNA extraction kit employed for the isolation of microbial DNA may represent a relevant source of bias. In this context, an artificial microbial community was generated by pooling bacterial cells of six different genera commonly identified in the human respiratory tract (Table 1). This microbial mock community was processed with five different microbial DNA extraction kits commercially available, i.e. Allprep PowerViral DNA/RNA Kit (QIAGEN), PureLink^TM^ Microbiome DNA Purification Kit (Invitrogen), QIAmp DNA Mini Kit (QIAGEN), QIAamp Fast DNA Stool Mini Kit (QIAGEN), and ZymoBIOMICS DNA Miniprep Kit (Zymo Research). These DNA extraction kits are methodologically comparable and do not include host DNA depletion, showing no technically significant differences.

DNA extracted from the microbial mock community through each microbial DNA extraction kit was quantified through Qubit Assay, revealing that the amount of DNA extracted appears to be largely influenced by the DNA extraction kit used (Table S1). In detail, Allprep PowerViral DNA/RNA Kit revealed the highest DNA extraction performance, followed by ZymoBIOMICS DNA Miniprep Kit and QIAmp DNA Mini Kit (Table S1). Conversely, QIAamp Fast DNA Stool Mini Kit and PureLink^TM^ Microbiome DNA Purification Kit showed the lowest capability to extract DNA (Table S1).

In order to identify possible correlations between DNA quantification and the predicted microbial composition, the five DNA samples were subsequently submitted for shotgun sequencing (Table S1 and Table 2). The standard library preparation protocol for Illumina shotgun sequencing was followed, and all the samples matched the minimum requirement of about 0.2 ng of DNA per µl. Notably, mock communities’ shallow shotgun metagenomic analysis revealed a comparable taxonomical profile (Table 2), confirmed through an intraclass correlation (ICC) analysis (average measures ICC = 0.98, single measures ICC reported in Table 2). Notably, Allprep PowerViral DNA/RNA Kit and QIAamp Fast DNA Stool Mini Kit exhibited the lowest accuracy in revealing *Streptococcus mutans* (relative percentage change >2500%), probably indicating a slightly lower accuracy of these two extraction kits. Nevertheless, these results suggest a general independent correlation between extracted DNA quantification and predicted microbial composition, when the amount of DNA retrieved is sufficient for sequencing library preparation and the number of reads is higher than 10,000, as reported previously for shallow shotgun approaches [27]. Moreover, to mimic the pulmonary niche and possible human cell contamination, we added a specific amount of eukaryotic DNA to the bacterial DNA mock communities, i.e. 106 MRC-5 human lung fibroblast cells. Additionally, the bacterial DNA mock communities have been used with two different concentrations, i.e. 10^4^ and 10^6^ cells, to simulate possible different bacteria cell loads present in the respiratory tract environment [29] and thus to better mimic the impact of human cell contamination on the bacterial community composition. As expected, the shallow shotgun metagenomic analysis based on the DNA extracted from the two treated microbial mock communities through each microbial DNA extraction kit revealed a higher presence of eukaryotic DNA in the mock with a concentration of 10^4^ bacterial cells (Table S1). However, the taxonomic analysis does not highlight significant differences based on the initial bacteria cell load or microbial DNA extraction kit (average measures ICC = 0.93, single measures ICC reported in Table S2) (Table S2), confirming a general independent correlation between initial bacterial load and/or extracted DNA quantification and predicted microbial composition.

#### Validation of the protocol through processing of human biological samples

In addition to the DNA extraction kit employed, the sampling method also represents a critical choice in disentangling the microbial community composition of human body sites [30]. For this reason, we tested the performances of the most widely applied microbial sampling protocols employed in respiratory tract-related studies, i.e. sputum collection as well as nasopharyngeal and oropharyngeal swabs. Moreover, the biological sample obtained for each of the three tested sampling approaches was processed using the five DNA extraction kits tested in this study.

In detail, seven individuals not affected by COVID-19 and without respiratory symptoms (Tables S3) underwent saliva and nasopharyngeal and oropharyngeal swabs collection, each repeated five times. Each collected biological sample was subsequently submitted to DNA extraction employing the five different DNA extraction kits (Table S1). Notably, sampling of the same individual multiple times was required to confirm the repeatability of the procedure and to overcome the modest bacterial load in the swab samples.

**Figure 1.**
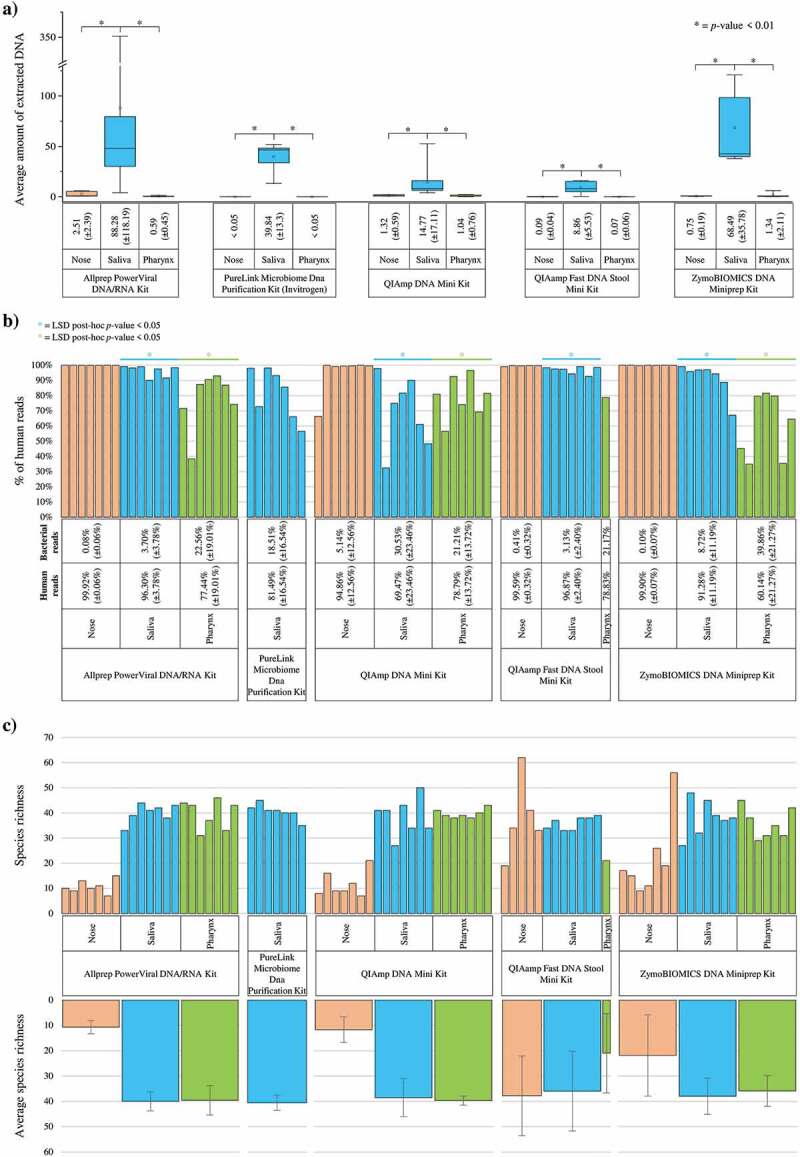
**Evaluation of the performances achieved by different commercial available DNA extraction kits**. Panel a shows the Whiskers plot representing the average amount of extracted DNA from the samples included in the study and treated with the different DNA extraction kits. The y‐axis reports the average amount of extracted DNA, while the x‐axis indicates the different DNA extraction kits and different sampling methods. The boxes are determined by the 25th and 75th percentiles. The whiskers are determined by 1.5 IQR (Interquartile range). The line in the boxes represented the median, while the square represents the average. Panel b reports a bar blot indicating the percentage of reads associated with eukaryotic sequences based on the DNA extraction kits and sampling methods. Panel c indicates bar plots regarding the species richness of each sample sequenced and the average, basing on the DNA extraction kits and sampling methods.

DNA extracted from each sample was quantified through Qubit Assay, revealing that the amount of DNA extracted appears to be influenced by the methodology of collection and the DNA extraction kit used (Figure 1a). In particular, DNA extraction from samples collected through swabs results in a markedly lower amount of DNA compared to saliva (p-value <0.01) (Figure 1a).

Regarding the DNA extraction kit employed, while no significant differences seem to be found between the commercial extraction kits used when processing sputum samples (Figure 1a), the PureLink^TM^ Microbiome DNA Purification Kit, and the QIAamp Fast DNA Stool Mini Kit retrieved the lowest quantity of DNA (<0.1 ng/µl) from the nasopharyngeal and oropharyngeal swabs respect to other kits. Remarkably, quantification cannot distinguish between eukaryotic and prokaryotic DNA, thus the whole DNA extracted from the samples was submitted to shotgun metagenomics sequencing.

#### Shotgun DNA sequencing and impact of DNA extraction kit on the reconstructed taxonomic profiles

All the DNA samples were submitted to the standard library preparation as described above. Notably, samples with DNA quantification below a quarter of the minimum requirement of 0.2 ng/µl indicated by the protocol mentioned above, i.e. 0.05 ng/µl DNA, were not further processed (Table S1). The latter encompasses a total of 22 samples that could not be sequenced (Table S1), which include only samples from nasopharyngeal and oropharyngeal swabs extracted through PureLink^TM^ Microbiome DNA Purification Kit and the QIAamp Fast DNA Stool Mini Kit, suggesting the low efficiency of these two extraction kits for these two specific sampling methods.

Shotgun sequencing of 83 selected samples produced an average of 81,538 ± 39,913 reads, ranging from 179,525 to 24,775 (Table S1). The raw sequencing reads were then mapped to the human genome in order to evaluate the percentage of host’s DNA extracted from each sample (Figure 1b). In detail, the QIAamp DNA Mini Kit, which did not provide the highest yield of DNA extracted (Figure 1a), encompassed the lowest amount of human DNA contamination in the saliva samples, with an average percentage of microbial DNA of 30.53% ± 23.46% (ANOVA *p*-value <0.05) (Figure 1b and Table S4). In contrast, ZymoBIOMICS DNA Miniprep Kit revealed the best performances in terms of yield of human DNA contamination in the oropharynx swabs with an average percentage of microbial DNA of 39.86% ± 21.27% (ANOVA *p*-value <0.05) (Figure 1b and Table S4). Thus, the host-filtering data underlined that the sole quantification of extracted DNA might be misleading for evaluating the performances in subsequent microbiota analyses. In fact, high host DNA contamination can drastically reduce the number of bacterial reads available for taxonomic and functional profiling.

Regarding the sampling methods tested, all nasopharyngeal samples showed a high abundance of eukaryotic contamination, representing in most cases 99% of the DNA sequenced (Figure 1b and Table S3). This result allowed obtaining a limited number of reads for the metagenomic analysis and precluded achieving reliable results, suggesting that this sampling method is not optimal to collect enough DNA from swab samples for shotgun metagenomics sequencing.

Moreover, after removing the host’s DNA, the datasets were submitted to species-level taxonomic profiling through the METAnnotatorX2 software [27] (Table S4). The profiling data obtained was used to evaluate the biodiversity of each sequenced sample expressed as species richness (Figure 1c). Remarkably, the biodiversity observed resulted independent of the five different DNA extraction kits employed (ANOVA and LSD post-hoc *p*-value >0.05) (Figure 1c).

**Figure 2.**
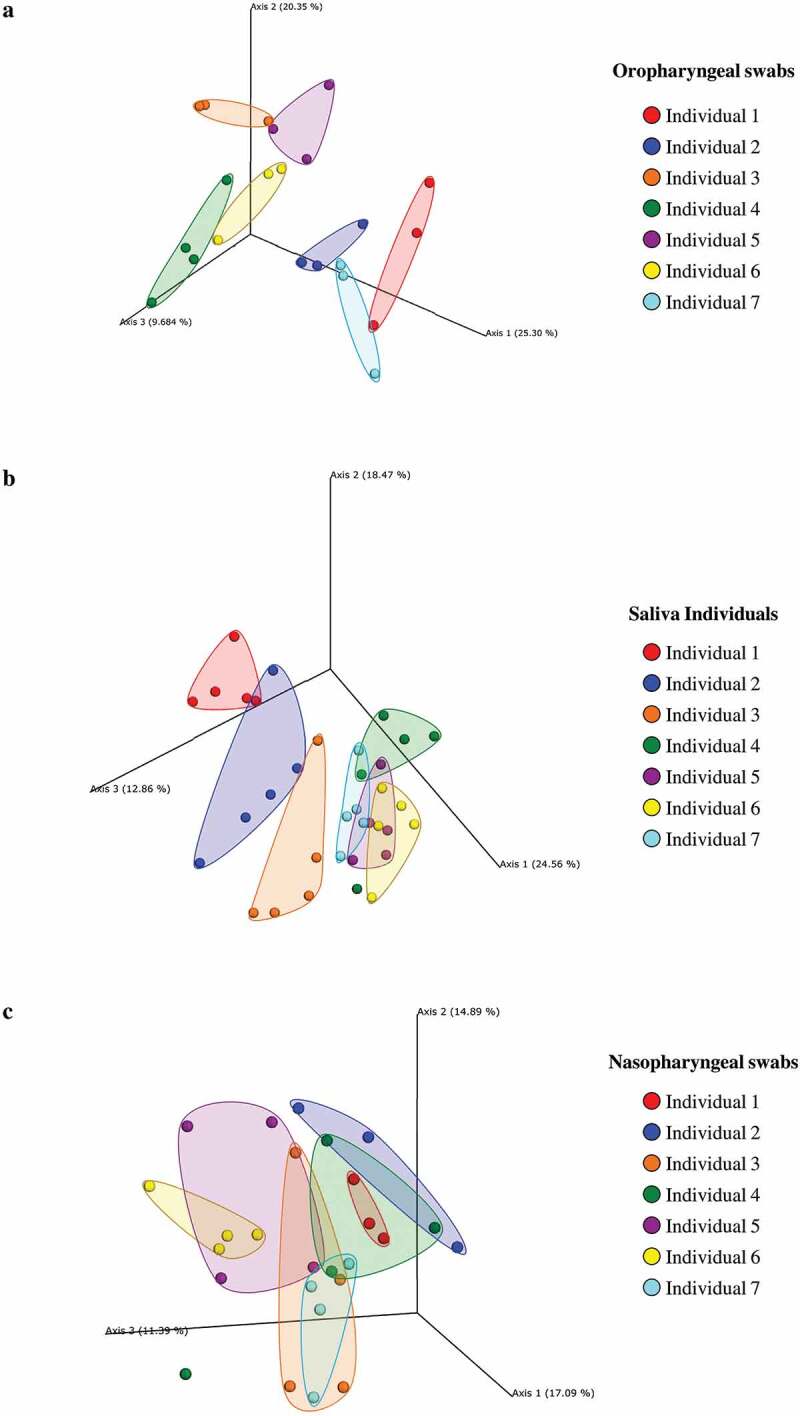
**Comparison of the taxonomic profiles of each extracted sample with the different extract kits**. Panel a shows the principal coordinate analysis (PCoA) of the samples obtained through oropharyngeal swabs. Each group includes a specific sample of an individual extracted with a specific DNA extraction kit. Panel b displays the PCoA of the saliva samples. Each group includes a specific sample of an individual extracted with a specific DNA extraction kit. Panel c reveals the PCoA of the samples obtained through nasopharyngeal swabs. Each group includes a specific sample of an individual extracted with a specific DNA extraction kit.

To investigate these results at the species level, we employed a PCoA analysis to compare the taxonomic profiles retrieved for the different extract kits in order to evaluate discrepancies in terms of bacterial species detected (Figure 2). The results revealed that datasets obtained from processing of the same oropharyngeal swab and saliva sample with different commercially available DNA extraction kits tend to cluster together, although we observed expected limited discrepancies in taxa abundance profiles, which can be attributed to the different performances in the cell lysis and DNA extraction (Table S1). In contrast, multiple nasopharyngeal swabs obtained from the same individual revealed high variability in taxonomic composition when processed with different DNA extraction kits (Figure 2), which can be imputed to the high fluctuations in the bacterial population composition retrieved from multiple sources sampling. Moreover, the nasopharyngeal appears to be highly contaminated by bacteria normally found on exposed skin or environmental samples, such as the species *Cutibacterium acnes* (>80% of the samples) and *Corynebacterium accolens* (>65% of the samples) (Table S5).

## Discussion

This study aimed at evaluating the best methodologic approach for the analysis of the upper airways’ microbiome due to its increasing relevance for the possible correlations with the clinical course of acute respiratory infections, including COVID-19. Here, we investigate in detail the accuracy of the most commonly applied sampling methods, i.e. oropharyngeal and nasopharyngeal swabs, as well as saliva collection, and the reliability of the microbial DNA extraction procedures, i.e. the common commercial kits employed for microbial DNA extraction from human biological samples.

Overall, the analysis of specific mock communities and the human respiratory biological samples revealed major discrepancies in total extracted DNA as well as human/bacterial DNA ratio and the observed microbial taxonomic profiles. In detail, based on all the results collected in this study, the QIAmp DNA Mini Kit and ZymoBIOMICS DNA Miniprep Kit represent the best options overall in terms of amount of DNA extracted, host DNA contamination, and downstream data analysis. Moreover, these data also revealed that oropharyngeal swab and saliva sampling should be preferred with respect to nasopharyngeal swabs in terms of reproducibility and host DNA contamination carry over.

## Conclusion

In conclusion, the results obtained in this study based on the oropharyngeal, as well as nasopharyngeal swab, and saliva samples highlighted different DNA extraction performance achieved with the common commercial kits. Moreover, the analysis of the three different sampling methods suggested that the nasopharyngeal swabs possess lower reproducibility and host DNA contamination capabilities.

## Supplementary Material

Supplemental Material
