# Supplementary material for: Guideline for the analysis of the microbial communities of the human upper airways
Source: J Oral Microbiol. 2022 Jul 28;14(1):2103282. doi: 10.1080/20002297.2022.2103282 (PMC9341376; doi:10.1080/20002297.2022.2103282)
Supplement: Supplemental Material [file ZJOM_A_2103282_SM7213.zip › Spplementary files/supplementary_information_2_no_authors (1).docx]

**Guideline for the analysis of the microbial communities of the human upper airways in COVID-related studies.**

**Supplementary information**

**Additional files**

**Table S1.** Data relate to the samples sequenced in this study.

**Table S2.** Bacterial profile at species level of the mock communities supplemented with eukaryotic cells.

**Table S3.** Metadata regarding the volunteers collected in this study.

**Table S4.** Percentage of eukaryotic and prokaryotic DNA in each sample analysed.

**Table S5.** Bacterial profile at species level of the samples included in this study.
